# Impacts of Deforestation on Childhood Malaria Depend on Wealth and Vector Biology

**DOI:** 10.1029/2022GH000764

**Published:** 2024-02-28

**Authors:** Tafesse Kefyalew Estifanos, Brendan Fisher, Gillian L. Galford, Taylor H. Ricketts

**Affiliations:** ^1^ Gund Institute for Environment University of Vermont Burlington VT USA; ^2^ Rubenstein School of Environment and Natural Resources University of Vermont Burlington VT USA; ^3^ Center for Environmental Economics and Policy UWA School of Agriculture and Environment The University of Western Australia Perth WA Australia

**Keywords:** Africa, *Anopheles gambiae*, *Anopheles funestus*, deforestation, land use change, malaria prevalence, vector ecology, poverty

## Abstract

Ecosystem change can profoundly affect human well‐being and health, including through changes in exposure to vector‐borne diseases. Deforestation has increased human exposure to mosquito vectors and malaria risk in Africa, but there is little understanding of how socioeconomic and ecological factors influence the relationship between deforestation and malaria risk. We examined these interrelationships in six sub‐Saharan African countries using demographic and health survey data linked to remotely sensed environmental variables for 11,746 children under 5 years old. We found that the relationship between deforestation and malaria prevalence varies by wealth levels. Deforestation is associated with increased malaria prevalence in the poorest households, but there was not significantly increased malaria prevalence in the richest households, suggesting that deforestation has disproportionate negative health impacts on the poor. In poorer households, malaria prevalence was 27%–33% larger for one standard deviation increase in deforestation across urban and rural populations. Deforestation is also associated with increased malaria prevalence in regions where *Anopheles gambiae* and *Anopheles funestus* are dominant vectors, but not in areas of *Anopheles arabiensis*. These findings indicate that deforestation is an important driver of malaria risk among the world's most vulnerable children, and its impact depends critically on often‐overlooked social and biological factors. An in‐depth understanding of the links between ecosystems and human health is crucial in designing conservation policies that benefit people and the environment.

## Key Points

- Although deforestation has been linked with malaria risk, little is known about whether socioeconomics or vector ecology moderate this effect
- Across six sub‐Saharan African countries, deforestation increased malaria risk in poorer communities more than in wealthier communities
- Understanding ecosystems‐human health‐poverty linkage is crucial in designing conservation policies that provide equitable health outcomes

## 1 Introduction

Global change due to human activity has modified global land cover drastically, having altered more than 75% of the ice‐free land worldwide due to human residence and land use (Ellis & Ramankutty, 2008; Ellis et al., 2021). Changes to forest cover are particularly evident in tropical areas, between 1980 and 2000 over 80% of new agricultural land came at the expense of tropical rainforests (Gibbs et al., 2010), and this progressed over time with an estimated loss of 2,100 km^2^ of tropical forest annually from 2000 to 2012 (Hansen et al., 2013). In West Africa, for example, deforestation, or forest loss, has led to a decline in natural vegetation cover and substantial gains in croplands (by 108%) and settlements (140%) between 1975 and 2013 (Barnieh et al., 2020). Such landscape transformations could lead to significant losses in biodiversity (Barlow et al., 2016) and changes in the abundance and composition of species, including species that are disease vectors (Loaiza et al., 2017; Newbold et al., 2015).

In addition, deforestation and subsequent land use changes alter human health risk factors for vector‐borne diseases in tropical areas (Chaves et al., 2018; Gottdenker et al., 2014; Guo et al., 2019). For example, alteration of natural habitat due to expansion of human population and increased urbanization, construction of roads and dams, and irrigation agriculture modify interactions among land, people, and disease vectors and their animal hosts, which affect the (re)emergence of vector‐borne diseases such as malaria (Jones et al., 2013; Lambin et al., 2010). Land use changes in Africa often also involve water bodies, which can create conditions favorable for the survival of vector species and thus could increase the incidence of malaria (Sheela et al., 2017). In addition, deforestation may increase human‐vector contact through environmental and demographic changes (Franklinos et al., 2019).

Malaria is a major public health concern worldwide: there were an estimated 229 million cases and 409,000 deaths in 2019 (WHO, 2020). Children under 5 years old represented 67% of the deaths globally and 94% of all malaria cases and deaths in sub‐Saharan Africa (SSA) (WHO, 2020). The three primary Anopheles vector species in Africa, commonly referred as dominant vector species (DVS)—*Anopheles gambiae*, *Anopheles arabiensis*, and *Anopheles funestus*—are largely responsible for transmitting the deadly cases in Africa (Sinka et al., 2010, 2012). Malaria has often been considered as a disease of poverty, as it has had a disproportionate impact on the poor (Schwartz et al., 2021; Tusting et al., 2016). However, the risk of malaria is also attributable to environmental factors driven by local land use practices (Kweka et al., 2016), proximity of water bodies or shoreline locations to households (Endo & Eltahir, 2018) and changes in climate and global processes (Caminade et al., 2019; Franklinos et al., 2019).

Deforestation and land use for agricultural activities alter malaria vectors' population dynamics and malaria risk (Austin, 2013; Burkett‐Cadena & Vittor, 2018). Several potential pathways have linked deforestation to human malaria and involved ecological risk at local scales (Tucker Lima et al., 2017). First, clearing forests can change environmental conditions, for example, by increasing sunlight and local air and water temperatures. These conditions influence the growth rate, larval development, and adult survival of vectors by creating ideal breeding sites and increasing adult productivity (Afrane et al., 2012; Kilpatrick & Randolph, 2012; Kweka et al., 2016). In Western Kenya, the productivity of *An. gambiae* was significantly higher in farmland and puddles than in associated natural forest or wetland habitats (Ndenga et al., 2011). Deforested areas, in comparison to forest areas, showed increases in the density and biting behavior of anopheline, such as *An. gambiae* and *An. funestus*, leading to higher vectorial capacity (Burkett‐Cadena & Vittor, 2018; Janko et al., 2018). Second, deforestation and forest fragmentation often create smaller forest patches and degraded forest edge habitats (Multini et al., 2020), increasing mosquito vectors abundance (Chaves et al., 2018) and the contact rate among infected mosquitos and susceptible human hosts and are driving spillover effects as mosquitos obtain blood meal from nearby human hosts (Faust et al., 2018; Hawkes et al., 2019).

In addition to the ecological mechanisms described above, several socioeconomic and demographic pathways link deforestation to malaria transmission (Baeza et al., 2017; Gottdenker et al., 2014). Deforestation often attracts humans for development activities such as agriculture, road building, mining, and logging (Hahn et al., 2014; Mitchell et al., 2022). In addition, in tropical countries including SSA, forest degradation and deforestation have been linked to poverty through selective logging, mining, and nontimber forest product overharvesting where rural people and migrants may colonize the “forest frontier” as a source of new agricultural lands (Sunderlin et al., 2005; Wunder, 2001). Human in‐migration increases the migrants' exposure to vectors, and the migrants may have low immunity to a particular vectors and inadequate health services. This situation is so common that the condition is known as “frontier malaria” (de Castro et al., 2006). Socio‐ecological changes coupled with changed malaria dynamics also affect people who live in or near malaria endemic areas (Wesolowski et al., 2012). Land use change follows successional stages of temporal land transitions, with the initial stage being associated with higher malaria risk because of slower economic development and later stages being associated with either a decline or a further enhancement of malaria risk (Baeza et al., 2017; Gottdenker et al., 2014).

Despite plausible links between deforestation and malaria, existing evidence about this relationship is mixed and contradicting across geographic regions (Hoffman‐Hall et al., 2020; Tucker Lima et al., 2017; Valle & Clark, 2013). Deforestation is associated with increased malaria prevalence in South America (Kar et al., 2014; MacDonald & Mordecai, 2019; Vittor et al., 2009). This trend, however, is the opposite in Southeast Asia, where the main vector depends on forest habitats (Garg, 2019). Studies in Africa showed inconsistent results, with malaria prevalence positively associated with deforestation in some cases (Berazneva & Byker, 2017), and no evidence of such relationship in others (Bauhoff & Busch, 2020). In most SSA countries, deforestation is largely driven by the steady expansion of rainfed and irrigated smallholder agriculture and small‐scale mining and logging may increase childhood malaria in both rural and urban contexts (Mitchell et al., 2022; Shah et al., 2022). In contrast, natural vegetation can potentially reduce malaria in rural areas (Shah et al., 2022).

Such inconsistent empirical findings have many potential causes, two of which we highlight here: poverty and vector biology. First, malaria and poverty are both common and linked to each other in the tropical regions of many low‐ and middle‐income countries (Sarma et al., 2019). In a bidirectional way, poverty may promote malaria transmission, and malaria may cause poverty by reducing economic productivity and therefore overall economic development (Bonds et al., 2012). In contrast, in Africa reduced malaria risk is associated with economic growth and development (Tusting et al., 2013), and income patterns are highly suggestive of underlying biophysical drivers, such as living in regions with high malaria burden (Bonds et al., 2012). Despite increasing attention, the complex relationship between malaria, deforestation, and poverty remains poorly understood and requires further research to inform policy (Pattanayak et al., 2006). The second potential cause of inconsistent findings is that changes in vector biology due to deforestation may increase malaria risk (Yasuoka & Levins, 2007) favoring more efficient malaria vectors, namely *An. gambiae and An. funestus* over non vector species (Burkett‐Cadena & Vittor, 2018; Kweka et al., 2016). Despite the growing body of literature on the relationship between deforestation and malaria, a recent review showed that broad‐scale studies on the influence of vector biology remain limited (Tucker Lima et al., 2017).

In this study, we examine the interrelated impacts of deforestation, vector biology, and poverty on malaria prevalence in children in six malaria‐endemic countries of SSA. These countries are in ecological regions dominated by tropical and subtropical grasslands, savanna, and shrublands that have experienced high rates of deforestation in recent decades. We used the United States Agency for International Development (USAID)‐sponsored Demographic and Health Surveys (DHS) and remote sensing environmental data (e.g., deforestation) for nationally representative sampling clusters from three SSA regions. We linked these data with environmental variables estimated at the same DHS sampling points. Using multi‐level mixed‐effects models, we investigate the relationship between deforestation and malaria prevalence and analyze how this relationship varies with household wealth and vector biology, while controlling for relevant confounders, including population density, livestock density, temperature, and precipitation (Bauhoff & Busch, 2020; Degarege et al., 2019; Kreppel et al., 2020; Mutuku et al., 2011; Tusting et al., 2016; Yamba et al., 2023). Our study deepens our understanding of the relationship among land use change, vector biology, and humans (Tucker Lima et al., 2017).

## 2 Materials and Methods

### 2.1 Health and Environment Data

**Figure 1:**
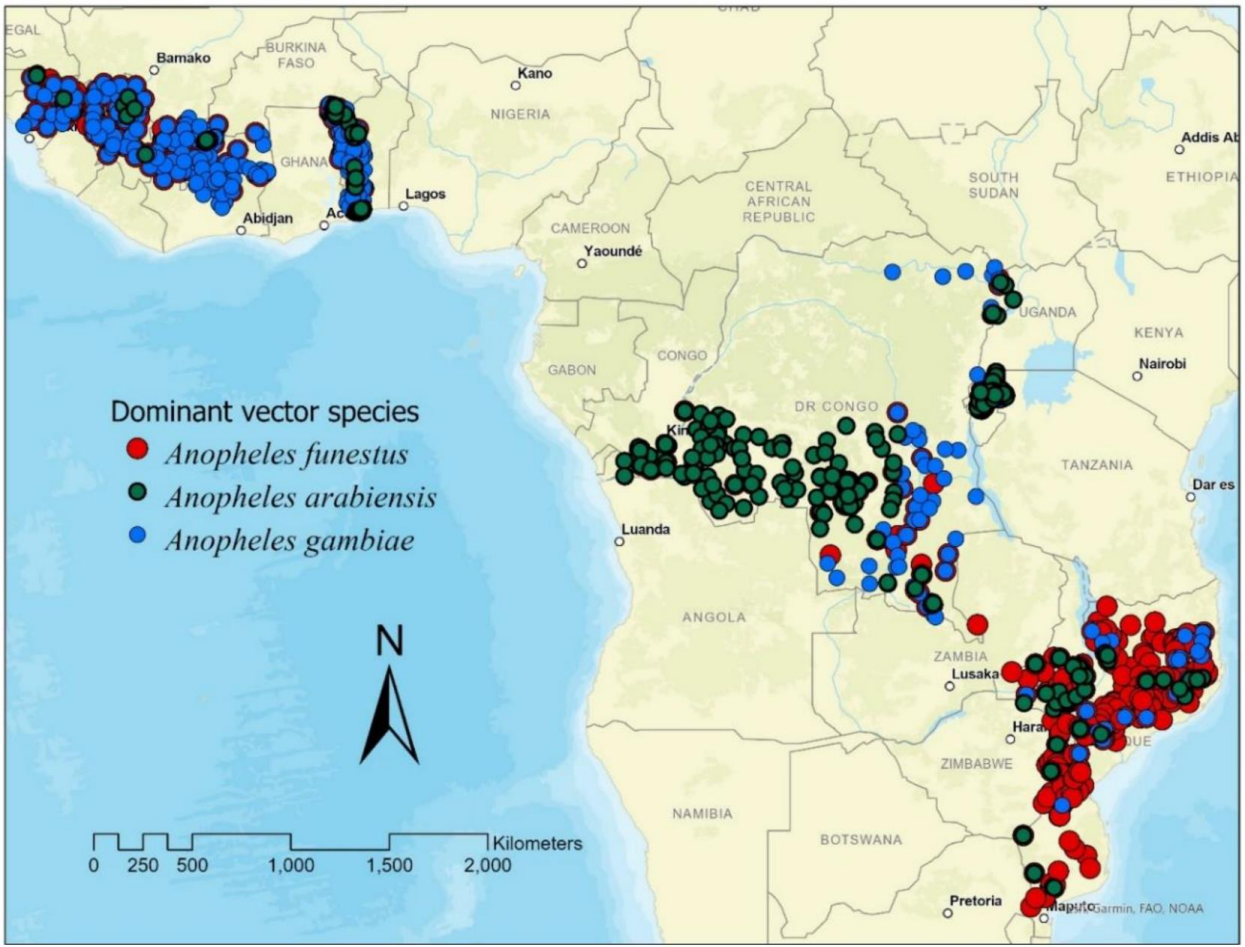
Map showing sampling clusters and distribution of the three dominant *Anopheles* species for Côte d’Ivoire, the Democratic Republic of the Congo, Guinea, Mozambique, Rwanda, and Togo, the six sub‐Saharan African countries included in this study.

We compiled rich data sets on health, demographic, socioeconomic, and environmental information for children living in six SSA countries (Côte d’Ivoire, the Democratic Republic of the Congo, Guinea, Mozambique, Rwanda, and Togo) spanning three regions: Central and Eastern Africa, Southern Africa, and West Africa (Figure 1). We obtained our dependent variable on health outcomes—the malaria prevalence in children under five—from USAID’s DHS surveys (ICF, 2017). The DHS data comprised health and demographic data from nationally representative household surveys conducted between 2010 and 2014. DHS surveys were administered using a multi‐stage sampling cluster survey design approach. Within clusters, households were randomly selected proportional to the population size. For those households and with parental consent, children under 5 years of age (hereafter referred as “children”) underwent blood sample tests for malaria parasite positivity (ICF, 2012). The DHS survey clusters were geo‐referenced, but to ensure confidentiality of respondents the cluster coordinates were displaced from up to 2 km (urban) to up to 5 km (rural), and a further 1% of rural cluster coordinates was displaced up to 10 km (ICF, 2012). We linked the DHS data with external remote sensing data of environmental and climate variables (Table S1 in Supporting Information S1 summarizes descriptions of the variables used in our analysis and their data sources). The full data set consisted of 11,746 children under 5 years from 8082 households in 1233 sampling clusters (Table S2 in Supporting Information S1).

#### 2.1.1 Malaria Prevalence

We defined malaria prevalence as the presence of parasitemia (malaria plasmodium parasites) based on lab blood tests under microscopy during the survey years. Using this information, we constructed a binary variable of malaria prevalence (1 = if the child was positive and 0 for negative). Although the DHS contained alternative measures of malaria prevalence, such as rapid diagnostic test (RDT) and fever episode, the lab blood test is considered a more reliable measure; and our use is consistent with previous studies on malaria and deforestation (Bauhoff & Busch, 2020). It is important to note that DHS surveys may be conducted during the dry season and outside the peak malaria transmission period, so malaria parasitemia data from surveys conducted outside peak transmission periods may not fully indicate peak transmission (ICF, 2012).

#### 2.1.2 Deforestation

We obtained temporal scale remote sensing data for the forest cover dominated by tropical and sub‐tropical grasslands, savannas, and shrublands biome for 1992/1993 (DeFries et al., 2000) and from vegetation/forest continuous fields for 2010/11 (DiMiceli et al., 2011). Forest was defined from the Vegetation Continuous fields as having greater than 40% tree cover according to The United Nations Environment Program's (UNEP) definition. Deforestation was computed as the proportion of changes in forest cover within 5 km buffer from cluster coordinates between 1992/93 and 2010/11. This buffer demarcation helps to account for daily movement and activities of people in malaria ‐frontier areas and to capture the spatial variability introduced by the intentional displacement of coordinates in the DHS surveys. Deforestation frontiers, including land uses and activities associated with malaria, can persist for several decades. We used definitions for tropical and subtropical grasslands, savanna, and shrublands that delineate woodland savannas (30%–60%) and forests (>60%) (Hansen et al., 2003) have been used previously for forest and woodland change studies in SSA (Galford et al., 2015; Potapov et al., 2012) (see detailed descriptions in Text S2 of the Supporting Information S1). Deforestation was defined as a change from forest cover to other forms of land use (Tucker Lima et al., 2017), which we estimated by a change over time in the continuous field greater than −5% (loss) for any given pixel. Net loss was summed across all pixels in a 5‐km buffer for each cluster, resulting in a deforestation estimate for each cluster (Figure S2 in Supporting Information S1).

#### 2.1.3 Mosquito Species Occurrence

We acquired malaria vector occurrence spatial data for Africa’s three DVS, *An. gambiae*, *An. arabiensis*, and *An. funestus* from the Malaria Atlas Project (MAP) (www.malariaatlas.org). The data set in the MAP predicts the probability of species occurrence (within ∼5 km buffer), ranging from 0 to 100 (Wiebe et al., 2017). This species distribution data considered combinations of environmental variables that best support *Anopheles* species presence in Africa. This spatial data was overlaid with DHS data using ArcGIS Pro (ESRI, 2020) for cluster buffer. For each vector species, a binary variable was used for the probability of occurrence if greater than 50 per cent considered for presence (=1), and 0 otherwise. This vector biology indicator was assigned for each DHS survey cluster. A previous study that assessed ecological zones’ suitability and distribution of *Anopheles* spp in Nigeria used a similar approach (Akpan et al., 2018).

#### 2.1.4 Demographic and Socioeconomic

We included human population density to control for its effect, given that land conversion for human use continues and this ongoing process may modify malaria transmission patterns (MacDonald & Mordecai, 2019). Human population density were also found to be associated with the risk of malaria (Moffett et al., 2007). We used globally gridded human population density data collected from the Center for International Earth Science Information Network (CIESIN) (CIESIN, 2016). We used the computed average value in a 10‐km buffer around each DHS cluster. We also acquired livestock density data from the Global Agro‐ecological Zones (GAEZ v3.0), Laxenburg, Austria (FAO/IIASA, 2010). Inclusion of livestock density data in our analysis helps to control for the potential of livestock to serve as alternative blood meal hosts, thus, diverting mosquitoes away from humans (Donnelly et al., 2015). Both population density (number of individuals per km^2^) and Tropical Livestock Unit (TLU, per km^2^) were assigned for each DHS cluster. We used a household wealth metric, a composite measure of a household's living standards based on the household's ownership of selected assets, dwelling characteristics, type of drinking water sources, toilet facilities, and other characteristics related to wealth status including agricultural land size and animals owned, from the DHS surveys' Wealth Index (Croft et al., 2018). We categorized the DHS‐Wealth Index into five levels of wealth quintiles: poorest, poorer, middle, richer, and richest. The index is a composite measure of poverty, not a single measure of socioeconomic status such as housing material, water supply source, sanitary condition, and health condition, which were used as control variables in previous studies (Bauhoff & Busch, 2020; Santos & Almeida, 2018; Yang et al., 2020).

Households in areas with higher malaria risks tend to undertake measures of behavioral avoidance. We constructed an indicator for insecticide‐treated bed net (ITN) use (hereafter referred as “*bed net use*”) to capture human behavior to control malaria vectors based on the DHS question about the “proportion of children who slept under ITNs the night before the survey.” We scored this intervention measure of bed net use with a binary variable (=1 if “some children” or “all children” slept under ITNs and 0 for “none” used an ITN). We also included a binary variable for residence (1 = rural vs. 0 = “urban” [baseline]). Rural populations are expected to experience higher levels of malaria transmission and severe disease than urban ones (Worrall et al., 2005). We included a child's age (in months) from the DHS surveys to control for the effect of age, so it would be detectable if malaria transmission were severe for younger children (WHO, 2020) or if malaria were positively associated with age (Afoakwah et al., 2018).

#### 2.1.5 Climate Data: Temperature and Precipitation

For temperature and precipitation, we used long‐term (1950–2000) mean monthly temperatures (in degrees Celsius, °C) and wettest quarter precipitation (millimeter, mm) data from the WorldClim data set (Hijmans et al., 2005) to control for their effect on malaria risk. Climate is defined as the pattern of historical weather observations. In this study, we controlled for historic climate variables to examine if climate relates to the transmission of malaria. Temperature and precipitation could influence the distribution and relative abundance of malaria‐vector mosquitoes and the associations between malaria incidence across malaria‐endemic countries in Africa, and rising temperatures in the future may impact malaria endemicity (Sinka et al., 2010; Wang et al., 2023). The current average annual temperature (°C) was calculated based on the long‐term Climate Hazards Group InfraRed Temperature with Station (CHIRTS) data set during the survey month and interpolated to generate 30 years of local time‐series data for each DHS cluster site. We also included the wettest quarter precipitation data (in mm) during the survey for each DHS cluster site. The wettest quarter precipitation was strongly correlated with annual precipitation (*r* = 0.6907). Furthermore, there were weak correlations between population density with temperature (*r* = −0.0957) and precipitation (*r* = −0.1163) while precipitation and temperature were moderately correlated in our study areas (0.3974).

### 2.2 Variable Screening and Multicollinearity

While examining the association between deforestation and malaria prevalence and the influence of wealth and vector biology, we controlled for the relevant confounders. We used standard techniques to address issues of omitted variables. *First*, our variable selection procedure is grounded on existing literature that examined environmental correlates of malaria endemicity and those with malaria‐deforestation linkage in SSA (e.g., Bauhoff & Busch, 2020; Weiss et al., 2015). *Second*, we addressed the issues of multicollinearity among our variables by calculating the variance inflation factor (VIF), which represents the amount of variability of a covariate explained by other covariates. The VIF of the suite of our covariates was not greater than 10, a generally accepted threshold value of exclusion of a variable (Salmerón et al., 2018). Finally, we standardized all the continuous variables to one standard deviation to help bring all predictors on to a common scale and help compare their relative importance to malaria prevalence.

### 2.3 Empirical Methods and Analysis

We used a multilevel mixed‐effects modeling approach that can fit and address the hierarchical nature of data to evaluate the association between deforestation and malaria prevalence after controlling for relevant variables. Our variables (demographic, socioeconomic, environmental, and climatic factors) are comprised of nested data with three levels: individual child nested within household, nested within clusters. The unit of analysis was the individual child, and the nested nature of the data leads to clustering, which imposes a correlation structure on the data. Thus, we applied three‐level, mixed‐effects models, accounting for the random effect of the two higher levels (household and cluster) to correct the biases in parameter estimates and provide correct standard errors and confidence intervals (Leyland & Groenewegen, 2020). The random intercepts at the household and cluster levels can capture the random effects at these two higher levels. Related studies relationships between land use change and malaria implemented a similar modeling approach (Mitchell et al., 2022; Shah et al., 2022). We present the full description of the empirical model in Supporting Information S1 (Text S1).

We conducted rigorous analysis through credible model specification and an informed approach based on literature about the intricate relationship between human‐environment. Specifically, our analysis involved three modeling approaches. First, we estimated general regional models to investigate the association between deforestation and malaria prevalence for three SSA regions. Second, we analyzed the effects of deforestation on malaria prevalence by wealth groups. For this, we split our sample population into five wealth quintiles and fitted five models separately, one each for the poorest, poorer, middle, richer, and the richest. Third, we split the sample data spatially into three based on the occurrence of the three DVS (*An. gambiae*, *An. arabiensis*, and *An. funestus*) and fitted three models separately, one for each, to examine their influence on the effects of deforestation. In the latter two approaches, we explored the presence of heterogeneity in the relationship between deforestation and malaria prevalence among different wealth levels and by differences in vectors biology. Odds ratios (ORs) were used to compare the effects of our main variables and covariates on malaria prevalence at 0.05 significance level. All analyses were carried out using Stata Version 17 with melogit command (StataCorp, 2022).

#### 2.3.1 Sensitivity Analysis

We conducted three sensitivity analyses to check the robustness of our results across different malaria prevalence measurements and modeling approaches. First, we examined an alternative specification by excluding the wealth quintile variables as controls from the main models to explore whether these variables affect by bed net use. The models showed no explanatory power of wealth levels over bed net use and supported the results of our main models by regions and by DVS (Figures S3 and S4 in Supporting Information S1). Similarly, we assessed the relationship between temperature, rainfall, and population density if there could be interactions between these factors. Then, we compared the findings of the models with and without climate variables and the findings in the model without climates variables hold the results of our main model (Figure S5 in Supporting Information S1). Second, using an alternative measure of malaria prevalence by rapid diagnostic test (RDT), we refitted all our models by wealth levels and by DVS (Figures S6 and S7 in Supporting Information S1). The main findings of our primary models hold true for both analyses, confirming the robustness of the estimates of our primary models. Third, we analyzed a subset of data that contained clusters dominated by a “single *Anopheles* species only” to avoid the effect due to vectors’ codominance by two or more dominant species in a sampling cluster (Figure S8 in Supporting Information S1). These sensitivity analyses confirmed the heterogeneity in the association between deforestation and malaria, suggesting the robustness of results from our main models.

## 3 Results

### 3.1 Malaria Prevalence and Sample Characteristics

We used a wide range of health, demographic, socioeconomic, and environmental data from six SSA countries—Côte d’Ivoire, Democratic Republic of the Congo, Guinea, Mozambique, Rwanda, and Togo—in three regions—Central and Eastern Africa (CEA), Southern Africa (SA), and West Africa (WA). Average malaria prevalence was 28.9%, varying widely from 55% in Guinea to 1.8% in Rwanda (Table S3 in Supporting Information S1). Average deforestation between 1992/93 and 2010/11 was 19.7%, with Mozambique experiencing the highest (50%), and Togo the least (2.3%) (Table S3; Figure S1 in Supporting Information S1). Malaria prevalence and deforestation varied by household wealth quintiles (Figure S2; Table S4 in Supporting Information S1). Malaria was more prevalent among the poorest (40.4%) and the poorer (36.7%) quintiles and least prevalent among the richest (6.2%). Similarly, deforestation was greater in clusters where the poorest and poorer households were located (20.5% and 24.9%, respectively) than in areas where the richest households were located (6.8%). Three dominant vector species (DVS) dominated our study areas, with *An. gambiae* occurring in most clusters (68.5%), followed by *An. funestus* (43%) and *An. arabiensis* (35.7%). The full descriptive summary of the variables is presented in Table S3 of the Supporting Information S1.

### 3.2 Effect of Deforestation on Malaria Prevalence

**Figure 2:**
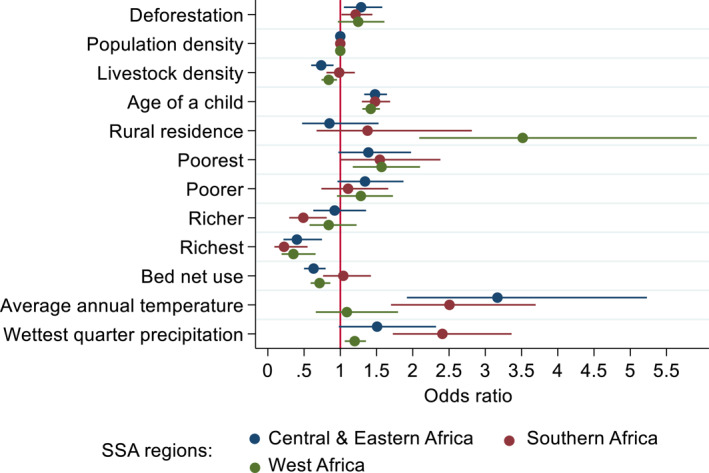
Association between deforestation and malaria prevalence by sub‐Saharan African (SSA) regions. The strength and direction of the association are measured as odds ratios (circles) along with 95% confidence interval (horizontal lines). Odds ratios greater than 1 are associated with increased malaria prevalence. Odds ratios between 0 and 1 correspond to reduced malaria prevalence. The red vertical line shows odds ratios = 1 correspond to “no association.” Sample sizes: Central and Eastern Africa *n* = 4,872; Southern Africa *n* = 2,384; and West Africa *n* = 4,490.

We found that deforestation was associated with childhood malaria, after controlling for relevant confounding covariates (Figure 2; Table S5 in Supporting Information S1). This relationship varied across the three regions: deforestation was associated with increased malaria prevalence in Central and Eastern Africa (CEA) and Southern Africa (SA). For one additional standard deviation in deforestation, malaria prevalence increased by 21%–28.7% (Odds ratios (OR): CEA 1.28; 95% CI [1.04, 1.58], SA 1.21; 95% CI [1.02; 1.44]). However, in WA we found no evidence of significant association between deforestation and malaria prevalence (OR1.24; 95% CI [0.96, 1.61]). Our robustness check based on alternative models also suggested similar results and the main findings were robust (Figure S3 in Supporting Information S1).

Several demographic, socioeconomic, and climate variables were also associated with childhood malaria, and their effects broadly varied among regions (Figure 2; Table S5 in Supporting Information S1). Population density was not associated with malaria prevalence with no variation in all regions. This finding was not as expected. While conducting sensitivity analysis by categorical variables for different population density levels (in person per km^2^) (lowest or less than 50; low or 50–200; and higher or ≥200), our findings showed no significant difference of effect on malaria prevalence for the lowest and higher population density levels (Figure S10 in Supporting Information S1). Rural areas in WA showed significantly higher malaria prevalence than urban areas, but not in CEA and SA. Age of a child in all regional areas was positively associated with increased malaria. Higher livestock density and use of bed net were associated with substantial reduction in malaria prevalence in CEA and WA, but not in SA. Our finding of positive but no significant effect in SA was unexpected. This result could be because the composition of samples of bed net users varies by SSA regions with bed users in Mozambique constituted the least (33.7%) compared to those in Central and Eastern Africa (63%) and West Africa (42.3%), and the overall sample population (49%). In addition, the effectiveness of bed nets decreases with declining bed net use while there was also a greater level of insecticide resistance by the dominant anopheles vectors such as *An funestus,* which occurred in 95% of clusters in Mozambique (Table S3 in Supporting Information S1), has been a serious concern (Cuamba et al., 2010). Wealth in all regions was related to reduced malaria prevalence, whereas poverty (poorest) was associated with increased malaria for SA and WA. Annual temperature was associated with increased malaria prevalence in CEA and SA, and wettest quarter precipitation was associated with increased malaria prevalence in SA and WA.

### 3.3 Deforestation Effects Moderated by Wealth

**Figure 3:**
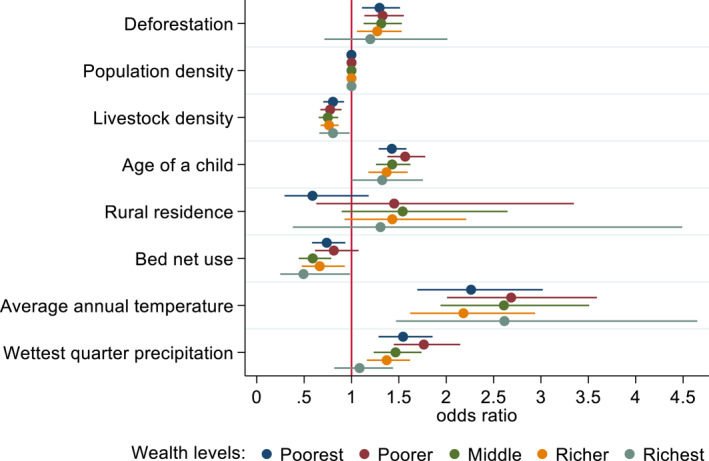
Effects of deforestation on malaria prevalence by wealth levels. The models for the five wealth quintiles (poorest (*n* = 3,090), poorer (*n* = 2,531), middle (*n* = 2,397), richer (*n* = 2,059), and richest (*n* = 1,669)) are represented by colored circles. The odds ratio represents the strength and direction of the association between deforestation and other variables (circles) along the 95% confidence interval (horizontal lines). Odds ratios greater than 1 are associated with increased malaria prevalence. Odds ratios between 0 and 1 correspond to reduced malaria prevalence. The red vertical line shows odds ratios equal to 1 that correspond to “no association.”

To understand how wealth affects the relationship between deforestation and childhood malaria, we disaggregated our data into sub‐population by household wealth levels. We found heterogenous effects of deforestation on malaria prevalence depending on wealth levels (Figure 3; Table S6 in Supporting Information S1). Specifically, deforestation had positive and significant association with malaria prevalence for the poorest (odds ratios (OR): 1.29, 95% CI [1.13, 1.53]), poorer (1.33, 95% CI [1.14, 1.55]), middle (1.31, 95% CI [1.14, 1.55]), and richer (1.27, 95% CI [1.05, 1.52]). That means, one additional standard deviation in deforestation increased malaria prevalence by 27%–33%. In contrast, we found no evidence of such a relationship in the richest quintile (OR 1.19, 95% CI [0.71, 2.01]). A separate sensitivity analysis based on a rapid diagnostic test (RDT) for an alternative measure of malaria prevalence confirmed these findings (Figure S6 in Supporting Information S1).

Our main wealth level models also suggested that demographic and climate variables were also associated with malaria prevalence. For all wealth groups, higher livestock density and bed net use (except for the poorer group) were negatively associated with malaria prevalence. The difference in results for the poorest and poorer wealth categories was not expected but was also a slight difference that was within the bounds of uncertainty in our model. One plausible mechanism for the unexpected result in our study setting where bed net usage was below 50%, could be non‐adherence among children, which could be associated with higher malaria (Rek et al., 2020). Furthermore, whereas annual temperature and wettest quarter precipitation were positively related to increased malaria prevalence for all the wealth groups, with the exception that wettest quarter precipitation did not increase malaria prevalence among the wealthiest.

### 3.4 Deforestation Effect Moderated by Vector Species

**Figure 4:**
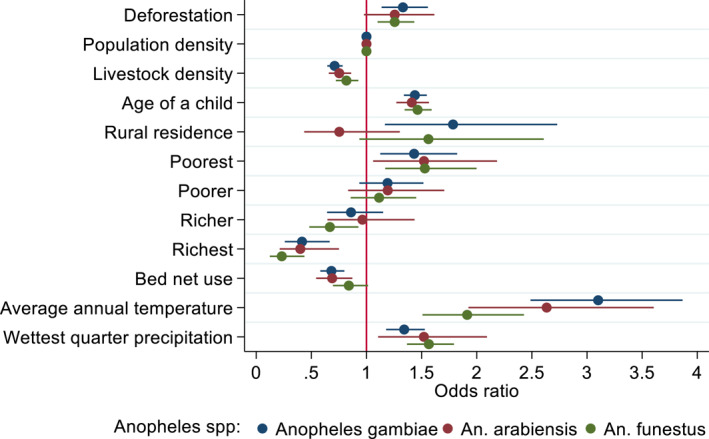
Effect of deforestation on malaria prevalence by dominant vector species (DVS). Models for the three DVS are shown in different colors: *An. gambiae* (*n* = 8,044), *An. arabiensis* (*n* = 4,195), and *An. funestus* (*n* = 5,052). The strength and direction of the association are measured as odds ratios (represented in filled color dots) along with the 95% confidence interval (horizontal lines). Odds ratios greater than 1 correspond to reduced malaria prevalence. Odds ratios between 0 and 1 correspond to reduced malaria prevalence. The red vertical line shows odds ratios equal to 1 that correspond to “no association.”

To evaluate how the relationship between deforestation and malaria prevalence differs based on vector biology, we conducted disaggregated analyses of our data set by the three dominant vector species (DVS): *An. gambiae*, *An. arabiensis*, and *An. funestus*. Our analysis showed that the effects of deforestation vary considerably by the DVS (Figure 4) Deforestation was associated with increased malaria prevalence in cluster areas where *An. gambiae* (OR 1.33, 95% CI [1.14, 1.55]) and *An. funestus* (OR 1.26, 95% CI [1.1, 1.43]) were dominant (Table S7 in Supporting Information S1 for coefficients). That is, a one standard deviation increased in deforestation increased malaria prevalence by 26%–33% in areas dominated by *An. gambiae* and *An. funestus*. In contrast, we found no significant association between malaria prevalence and deforestation in areas where *An. arabiensis* were dominant (OR 1.25, 95% CI [0.97, 1.62]).

When assessing confounders, age of a child, livestock density, wealth levels, bed net use and climate variables were associated with malaria prevalence in the DVS clusters. Rural areas with clusters dominated by *An. gambiae* were associated with increased malaria prevalence, but that relationship weakened in areas where the other two vectors dominated. Bed net use had negative effect in *An. gambiae* and *An. arabiensis* dominated areas but not for those areas with dominant *An. funestus*. While the poorest saw increased malaria prevalence across the three DVS, the richest showed the opposite result. Consistent patterns were found for annual temperature and wettest quarter precipitation, all of which had positive effects for all the three DVS.

Our sensitivity analyses based on alternative model specifications excluding wealth level confounders (Figure S4 in Supporting Information S1) and alternative measures of rapid diagnostic test (RDT) for malaria prevalence (Figure S7 in Supporting Information S1) supported the main findings suggesting the robustness of our primary model estimations. Additionally, analysis that considered cluster sites which were exclusively dominated by one of the three DVS (excluding codominance) showed consistent results and the findings varied among the three DVS models (Figure S8 in Supporting Information S1).

## 4 Discussion and Conclusion

This study reveals important new social and ecological dimensions to the relationship between deforestation and malaria. Using a unique data set combining demographic, health, socioeconomic, biological, and environmental data, we find that deforestation is associated with increased prevalence of malaria in children under 5 years of age. We also find that deforestation‐malaria relationships are strongest in the least wealthy communities, indicating that the poor are likely the most vulnerable to health risks from ongoing deforestation. The malaria‐deforestation relationship also differs among regions dominated by different but locally important mosquito vectors, which may help to explain the variation in previous studies regarding malaria risk in changing landscapes and across ecological regions in tropics (Fornace et al., 2021; Rice et al., 2021; Zohdy et al., 2016). These findings highlight the critical social and ecological factors that moderate the impacts of deforestation on infectious diseases, and they suggest that synergies between public health and conservation efforts are important (Bauch et al., 2015).

Overall, we find a positive relationship between deforestation and malaria prevalence across the three SSA study regions, suggesting that deforestation is an important environmental predictor of malaria risk (Figure 2). These findings are consistent with related studies that report deforestation's positive association with higher malaria prevalence in Africa (Berazneva & Byker, 2017) and Latin America (MacDonald & Mordecai, 2019; Vittor et al., 2009). These studies document that land use changes (e.g., deforestation) leading to the presence of humans where agriculture, and forest‐related activities are clear determinants of malaria risk. Our findings differ from a previous study in SSA by Bauhoff and Busch (2020), who used a larger data set from 17 SSA countries. Although these authors found no clear linkage, they hypothesized a presence of potential deforestation‐malaria relationship at a different scale of study, which our results support. Our study differs from Bauhoff and Busch (2020) in that we examined six SSA countries that experienced substantial forest cover loss over two decades and focused on specific biome types—tropical grasslands, savanna and shrublands ecological zones—where malaria is predominantly meso‐ or hyper‐endemic.

Beyond showing the effects of deforestation on malaria prevalence, this study provides evidence of the complex linkages between deforestation, malaria, and poverty. Previous studies suggest that malaria and poverty are closely related and document the presence of high malaria rates in SSA countries (Sarma et al., 2019). In the same geography, poverty and deforestation are considered major causes of malaria (Sachs & Malaney, 2002; Worrall et al., 2005), but their joint effects are rarely considered (Pattanayak et al., 2006). We show that the impact of deforestation on malaria prevalence varied by household wealth level. These findings suggest that different wealth levels moderate the deforestation‐malaria linkage, indicating malaria prevalence increases with deforestation for the poorest households, but not for the richest households (Figure 3).

This malaria risk could be attributed to the various determinants, including environmental, occupational, and human/social drivers (Schwartz et al., 2021). Here we discuss two potential mechanisms. *First*, poor households may constitute the majority of dwellers and migrants at the forest edge in areas where malaria vectors are abundant and malaria is more prevalent (Saker et al., 2004). In these frontier areas, deforestation and land use changes may increase human contact with new vectors and parasites (Faust et al., 2018) by providing additional breeding sites for anthropophilic *Anopheles* species and hence increasing the risk of malaria infection (Kweka et al., 2016). For example, paddies irrigation activities by less wealthy communities in the African highlands and desert fringes can increase vector density in areas of seasonal malaria transmission (Ijumba & Lindsay, 2001). Furthermore, the same socioeconomic conditions that lead people to migrate (e.g., poor housing; lack of access to clean drinking water, sanitation conditions, and bed nets; and lower education) tend to favor malaria transmission in deforested and newly colonized areas (Tusting et al., 2013; Yang et al., 2020).

*Second*, people living in poverty often are obliged to work in outdoor forest‐related activities (e.g., logging, clearing) that have a high rate of exposure to malaria vectors either through creating suitable breeding sites for larvae or increase contact with vectors (Schwartz et al., 2021). Previous research documents that in Burundi, the Democratic Republic of the Congo, Rwanda, and in many countries in South America, most poor people live in rural areas and work as subsistence farmers on relatively small plots of land, and many also enter artisanal mining on a seasonal basis (Hilson & Garforth, 2012). Outdoor work (e.g., agriculture activities at night) and sleeping in poor housing and temporary forest shelters such as tents may enhance malaria transmission through an increase in mosquito bites (Guyant et al., 2015; Smith et al., 2021). Generally, frontier deforestation coincides with an increase in malaria transmission at early stages of land transformation (Baeza et al., 2017). Areas characterized by ecologically degrading activities have the highest risk for the emergence of malaria (Jones et al., 2013). In the present study we find that less wealthy households live in areas where the deforestation rate was the highest (20.4%–24.9% for the poorest, poorer, and middle wealth levels), compared to the deforestation rate in areas of the wealthy (6.9% for the richest) (Figure S2 in Supporting Information S1). For these groups our analysis by wealth levels suggests that deforestation was associated with increased malaria prevalence. Deforestation may also reduce households' wealth and thus their capacity to invest in health care and pay for malaria prevention and treatment (Myers et al., 2013). National contexts differ, however. In rural Uganda, for example, there is evidence of decreasing malaria transmission and malaria risk in areas of greater socioeconomic development, which is attributed to success in agricultural development and lower indoor biting rates (Tusting et al., 2016). Our findings that deforestation does not impact malaria prevalence for the richest households suggest that households' higher socioeconomic status insulates children living within them. Overall, in SSA, the use of preventive and treatment interventions for malaria appears to be related to higher socioeconomic status, and lower socioeconomic status is more likely to increase vulnerability to malaria (Degarege et al., 2019).

Critical to malaria transmission in SSA is the presence of highly efficient *Anopheles* vector species (Sinka et al., 2012) and variations among these vectors are essential to understanding malaria transmission in a changing landscape (Fornace et al., 2021; Niang et al., 2019). In the present study, local DVS differentially modify the relationship between deforestation and malaria (Figure 4). Deforestation is associated with increased malaria risk in areas where *An. gambiae* and *An. funestus* are dominant, confirming that both are highly efficient vectors (Sinka et al., 2010). Deforestation favored these two as deforested sites offer suitable sites for breeding and survival, and consequently sustain local malaria transmission (Burkett‐Cadena & Vittor, 2018). In Kenya, the survival of *An. gambiae* was 55%–57% higher in deforested areas characterized by habitats fully exposed to sunlight and temperatures elevated by 3–4°C, as compared to 1%–2% temperature increases in forested areas (Tuno et al., 2005). *An. gambiae* and *An. funestus* are both highly anthropophilic (prefer human blood), endophilic and endophagic (i.e., rest and feed indoors), and these behaviors contribute to significant increase in malaria transmission (Githeko et al., 1996; Sinka et al., 2010). Such feeding ecology contributes to the higher vectorial capacity of the two vectors compared to *An. arabiensis*, which is mainly zoophilic (prefers livestock) and exophilic (rest outdoors) (Niang et al., 2019; Sinka et al., 2010).

This multi‐country analysis on the importance of vector biology is a substantial advance in understanding land use change and infectious disease dynamics (Fornace et al., 2021). Several finer‐scale lines of evidence support our findings and suggest that deforestation influences mosquito larvae survivorship and adult productivity and thus increases malaria risk (Rice et al., 2021; Yasuoka & Levins, 2007). In most SSA countries, deforestation and alternative land uses create suitable breeding sites for *An. gambiae* and *An. funestus*, influence their bionomics, including increasing density, entomological inoculation rates, and vectorial capacity (Niang et al., 2019; Yasuoka & Levins, 2007). Increased exposure to agriculture and rainfed cropland were associated with increased malaria risk in children under five across rural areas due to increased indoor biting rates among *An. gambiae* mosquitoes (Janko et al., 2018; Shah et al., 2022). *An. funestus* also may be an important vector in areas where vector control methods can effectively control *An. gambiae* (Wiebe et al., 2017). An*, funestus* also appears to extend wet season malaria transmission by *An. gambiae* into the dry season (Coetzee & Fontenille, 2004). By contrast, we find no evidence of association between deforestation and malaria risk in regions dominated by *An. Arabiensis*, suggesting its limited role in malaria transmission among humans in our context, likely due to its low anthropophilic rate and host‐shifting behavior with preference to feed on livestock blood meal (Chirebvu & Chimbari, 2016; Niang et al., 2019).

When evaluating the effect of demographic confounders, the result from our multicounty study showed that population density was not associated with malaria prevalence. This finding diverges from previous findings (Weiss et al., 2015). In Africa, although areas of high human population density were found to be associated with the higher risk of malaria, no clear pattern can be observed in the distribution across landscapes (Moffett et al., 2007). The findings in our case study could be attributed to context specific risk factors, and hence other relevant ecological and social factors driven by small‐scale land‐use variability and climate change could affect *Anopheles* spp. distribution and concomitant plasmodium infection in humans and mosquito vectors (Kweka et al., 2016; Ndenga et al., 2011). Malaria risk can be high in areas with increasing population density including in urban areas where exposure to vectors and malaria transmission intensity are high, have conducive environmental conditions and poor socio‐economic settings. In fact, the findings from our study suggest that rural residence in West Africa and temperature in Central and Eastern Africa were strongly associated with increased malaria prevalence (Figure 2). Previous studies also suggest non‐linear relationship between population density and malaria prevalence (Kabaria et al., 2017).

Our findings have broad implications for infectious disease dynamics and control within the wider socioeconomic and environmental contexts in which they occur. A growing body of evidence links deforestation with risks of infectious diseases like malaria. However, the effects of deforestation and subsequent land use impact on malaria (and other infectious diseases) are complex and context specific. They depend on interactions among environmental, social, economic, and ecological factors, including vector behavior, poverty, health infrastructure, and human behavior (Myers et al., 2013; Pattanayak et al., 2006; Schwartz et al., 2021; Shah et al., 2019).

To continue to advance our understanding of this complex system, studies of finer spatial and temporal resolution will be important. Given that small‐scale land use variability affects *Anopheles* spp distribution and malaria infection in humans (Zohdy et al., 2016), better understanding seasonal variations in environmental factors and transmission levels (including malaria peak seasons) using dynamical models and field data is critical. Unpacking these dynamics in landscapes under substantial human use (e.g., secondary growth forest and agricultural ecosystems) is particularly important because these ecosystems host a greater proportion of locally DVS than those in natural areas (Ferraguti et al., 2022; Janko et al., 2018; Kweka et al., 2016).

In conclusion, understanding the relationships among deforestation, socioeconomic factors, vector biology, and malaria risk is essential to addressing malaria dynamics. It can help identify populations at risk and design well‐targeted environmental interventions to control important vector species and help achieve better health outcomes at a landscape level. More generally, our results reinforce the fact that social, ecological, and health systems are tightly interconnected, such that efforts to reduce deforestation can improve human health and well‐being in malaria endemic tropical regions, including Africa.

## Conflict of Interest

The authors declare no conflicts of interest relevant to this study.

## Supporting information

Supporting Information S1

## Data Availability

USAID Demographic and Health Surveys (DHS) data and historical forest cover data (DeFries et al., 2000; DiMiceli et al., 2011) were used to analyze data for this manuscript. The DHS data is protected under the DHS Program data policy and access restrictions (e.g., authentication required), and only the DHS Program is authorized to provide access to the data available from ICF (2017). Additional data sets that are linked with DHS data set, including population density, livestock density, climate variables (long‐term annual temperature and precipitation of wettest quarter) compiled from other sources are described in this submission. Additionally, data on the vectors occurrence spatial data from the Malaria Atlas Project (MAP) is available at Wiebe et al. (2017). The geospatial data was overlaid with DHS data using ArcGIS Pro (ESRI, 2020) licensed to the University of Vermont. These data obtained from external sources outside of the DHS database are available and archived in figshare open access repository (Estifanos et al., 2024). Stata Version 17 (Stata SE17) Software (StataCorp, 2022) licensed to the University of Vermont was used for the analysis and for creating all figures included in this submission. All Stata codes for analysis can be within the Supporting Information S1 (Data Set S2) and is achieved in Figshare open access repository (Estifanos et al., 2024).
