# Supplementary material for: Impacts of Deforestation on Childhood Malaria Depend on Wealth and Vector Biology
Source: Geohealth. 2024 Feb 28;8(3):e2022GH000764. doi: 10.1029/2022GH000764 (PMC10902572; doi:10.1029/2022GH000764)
Supplement: Supplementary file 1 — Supporting Information S1 [file GH2-8-e2022GH000764-s001.docx]

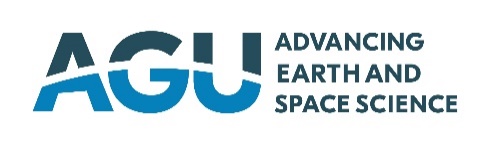


*GeoHealth*

Supporting Information for

**Impacts of deforestation on childhood malaria depend on wealth and vector biology**

# Tafesse Kefyalew Estifanos^1,2,3*^, Brendan Fisher^1,2^, Gillian L. Galford^1,2^, and Taylor H, Ricketts^1,2^

^1^ Gund Institute for Environment, University of Vermont, Burlington, VT, 05405, USA.

^2^ Rubenstein School of Environment and Natural Resources, University of Vermont, Burlington, VT, 05405, USA.

^3^ Center for Environmental Economics and Policy, UWA School of Agriculture and Environment, The University of Western Australia, 35 Stirling highway, Crawley, Perth, 6009 Western Australia

**Contents of this file**

Text S1 to S2

Figures S1 to S10

Tables S1 to S7

**Introduction**

This file presents a brief overview of the supporting information that are associated with the data, methodology and descriptive summary and ancillary analysis. The model and variables description along with the sources of the data is given followed by the descriptive summary of the data. The Demographic and Health Surveys, data was linked with remote sensing environmental data and spatial data from Malaria Atlas project (MAP) about the dominant vector species in Africa. The data is nested which was measured at different levels (individual child, household, and cluster). A stepwise selection of variables was implemented preliminary informed by literature A series of sensitivity analysis and robustness check results were presented at the end.

Text S1.

We used multilevel mixed effects logit models, also known as multilevel model (Leyland & Groenewegen, 2020) to analyze our data. These models were appropriate to analyze our nested data sets while accounting for the effect of covariates measured at three different levels (individual child, household, and cluster) in the context of nature-human health studies (Mitchell et al., 2022; Shah et al., 2022). We used the three-level mixed effect model specification with two random intercepts (at cluster level and household levels) to address the issue of clustering due to repeated observations and avoid the resulting parameter estimate biases. Our dependent variable was binary malaria prevalence (measure of malaria positivity) was represented as $y_{ijk}$, for child (i) living in household (j) and in cluster (k). The probability of malaria positivity (=1) is given as $P_{ijk}=Pr(y_{ijk}=$1) in a logit function. The three-level model can be written as:

$y_{ijk}= B_{0}$ +$\beta_{1}x_{ijk}+ u_{1jk}x_{ijk}+ v_{0k}+ u_{0jk}+ e_{0ijk}$

Where $y_{ijk}$ was the health outcome variable (malaria prevalence) for the ith unit at level individual child level (level 1) jth unit at household level (level two); $x_{ijk}$ was an observed explanatory variable for level one (e.g., age of a child); j indexes level two variables (e.g., wealth quintile levels, bed net use) and k refers to the variables at level 3 (e. g. deforestation, population density, livestock density, annual temperature, wettest quarter precipitation); $u_{0jk}$ and $v_{0k}$were the random intercepts for levels two and three, respectively. $u_{1jk}$was the random effect for $x_{ijk}$at level two. $\beta_{0}$ was the intercept. While $x_{ijk}$represented the explanatory variable, $\beta_{1}$ is its effect accounting for the random variation at levels two and three.

Text S2. Data description

This subsection describes the data and variables used in this study. Table S1 summarizes the list of variables and their description along with references of the various data sources.

**Demographic and Health Surveys**

Information on health, demographic and socioeconomic was obtained from Demographic and Health Surveys (DHS) database of the United States Agency for International Development (USAID) (ICF, 2017). Our DHS data comprised from a nationally representative household surveys conducted between 2010 and 2014 (Fig 1; *Table* S2). DHS were administered using a multi-stage sampling cluster survey design approach. Within clusters, households were randomly selected proportional to the population size from which children under five years underwent a blood sample tests (ICF, 2012). Our dependent variable for the health outcome, malaria prevalence, measured the presence of parasitemia under laboratory blood test for sampled children living in six SSA malaria endemic countries (Democratic Republic of the Congo, Cote d’Ivore, Guinea, Mozambique, Rwanda, and Togo) (**Appendix** Table S1). The DHS survey clusters were geo-referenced but to ensure the confidentiality of respondents the cluster coordinates were displaced from up to 2 km (urban) to up to 5 km (rural) with a further 1% of rural cluster coordinates displaced up to 10 km. DHS data was linked with remotely sensed environmental variables at a sampling cluster point representing a sampled population.

**Climate variables**

Mean monthly temperature at the time of the DHS survey was used to represent temperature effects on the presence of parasitemia (malaria presence), with greater temperatures expected to increase malaria presence. Rainfall (total) during the wettest quarter (season) of the year was used to represent the moisture conditions that foster *Anopheles* species’ breeding habitat. Mean monthly temperature (°C) and Wettest Quarter Precipitation (mm) were extracted from the WorldClim dataset for each DHS cluster sampling sites (Hijmans et al., 2005).

**Deforestation**

Six malaria endemic countries in SSA with ecological regions dominated by tropical and subtropical grasslands, savanna, and shrublands that have large extents of forest cover and also high rates of deforestation in recent decades. Deforestation frontiers last for decades; that is, the forest clearing activities, frontier habitats for mosquitos, socioeconomic conditions, and more may persist for 20 or more years. To consider the legacy of deforestation on malaria prevalence, we analyzed forest cover change for the period closest to the DHS survey (2010/2011) compared to forest cover in 1992/1993. Vegetation continuous fields (VCF) are one estimate of forest cover, where VCF is represented as a % and correlates to the type of tree cover. The MODIS VCF (250 m) definitions for tropical and subtropical grasslands, savanna, and shrublands, delineate woodland savannas (30-60%) and forests (>60%) (Hansen et al., 2003) and have previously used for forest and woodland change studies in SSA (Galford et al., 2015; Potapov et al., 2012). Here, we applied a conservative definition of forest or woodland cover used by UNEP of 40% or greater in VCF (DiMiceli et al., 2011). The MODIS record begins in 2000 so, to consider the deforestation activities since the 1990s, we use the predecessor data set to MODIS—AVHRR Continuous Fields (1 km). The MODIS data was designed to extend the record started by AVHRR, however, to compensate for any potential differences in VCF values between the two data products due to high frequency noise, native image resolutions or other factors, we only considered changes in VCF that exceeded -5% from 1992/1993 to 2010/2011 at a spatial resolution of 1km and 250 m, respectively. The VCF data was extracted for each 10 km buffer corresponding to a DHS cluster. The buffer accounts for both the uncertainty in the DHS coordinates and the effects of deforestation within a zone of influence (i.e., human movement to markets, schools, hunting/foraging areas). The net change in forest cover, or deforestation, was then used for each DHS cluster.

**Occurrence of Dominant Vector Species**

In sub-Saharan Africa the high level of malaria transmission is predominantly attributed to the presence of the most efficient vectors, namely, *An. gambiae*, *An. arabiensis*, and *An funestus* which are also considered as the dominant vectors species (DVS) (Sinka et al., 2010). The ecological difference among these species influence their role in malaria transmission (Wiebe et al., 2017). This data is compiled based on cases studies and extensive literature on the relationship between species occurrence and combinations of site-specific environmental covariates for the study countries. The MAP data holds the predicted probability of the species occurrence as raster data (~5km), which ranges on a scale of 0 to 1.0 for the three DVS. *An. gambiae, An arabiensis* and *An. funestus* (Wiebe et al., 2017). We extracted the relative probability of species presence at all locations within the species range and the data was assigned for each DHS survey clusters included in this study. This data was used to create presence-absence binary data for each species, and we considered 1 if the probability of occurrence greater than fifty per cent and 0 otherwise. This approach was consistent previous studies in Nigeria (Akpan et al., 2018).

**Human and livestock densities**

To control this, we obtained data on the population per square kilometer within 5 km of each cluster sites was also computed using a population density grid (CIESIN, 2016). The global gridded human population density data collected from the Center for International Earth Science Information Network (CIESIN) 2016. The gridded population of the world Version 4 (GPWv4) was adjusted to match 2015 Revision UN WPP Country Totals on Socioeconomic Data and Applications Center (SEDAC). This dataset provided a globally consistent and spatially explicit data for our use because it provided a spatially disaggregated population layer that is compatible with our demographic and health indicators information from DHS data sets.

Additionally, we included data on ruminant livestock density at cluster 10 km grid circa 2000 (FAO/IIASA, 2010). Livestock density data (expressed as Tropical Livestock Units per km2 (TLU/km2) was sourced from International Institute for Applied Systems Analysis (IIASA) and the Food and Agriculture Organization of the United Nations (FAO) that have been continuously developing the Agro-Ecological Zones (AEZ) methodology over the past several years for assessing agricultural resources and potential (FAO/IIASA, 2010). This data was nationally disaggregated into individual spatial units (cluster level) and were represented in different levels with livestock density values (Table S1). The categorical variables were assigned to each DHS cluster site.


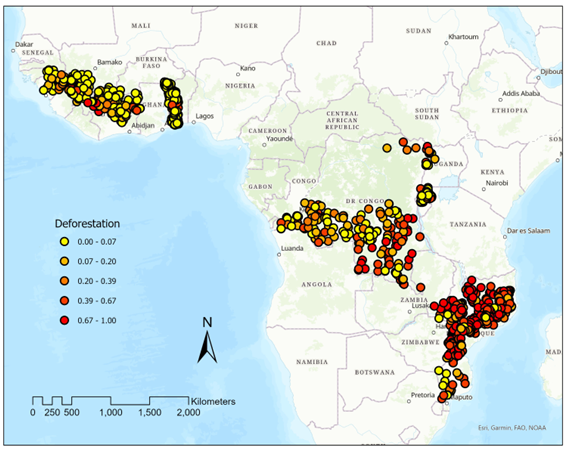


Figure S1. Deforestation for the study countries in sub-Saharan Africa (1992/93-2010/11). The dots represent sampling cluster sites, and the color shows the intensity of deforestation.

Figure S2. Summary of deforestation by wealth levels. Mean and confidence interval for each group of bar chart with error bars.

Figure S3. Sensitivity analysis of the effect on malaria prevalence by regions without wealth levels.

Figure S4. Sensitivity analysis of the effect on malaria prevalence by different dominant Anopheles vector species (DVS) (alternative model specification by excluding wealth levels).

Figure S5. Summary of models results by SSA regions without climate variables (a sensitivity analysis for the relationship between population density and climate variables in comparison with models with climate variables).

Figure S6. Sensitivity analysis of the effect on malaria prevalence (measured by alternative measures of rapid diagnostic test, RDT) by wealth levels.

Figure S7. Sensitivity analysis of the effect on malaria prevalence (measured by alternative measures of rapid diagnostic test, RDT) by different *Anopheles vector species*.

Figure S8. Sensitivity analysis summary on the effect of deforestation on malaria prevalence (blood laboratory test) based on by only one dominant vector species

Figure S9. General model including wealth and vectors species covariates. For wealth quintile levels (middle quintile is the base case) and for vector species (*Anopheles arabienesis* is the base case)

Figure S10. Association between deforestation and malaria prevalence by sub-Saharan African (SSA) regions (Sensitivity analysis on effect of population density by three levels (Baseline the lowest level 0-50 individuals sq. km-2)

Table S1. Summary of description of the variables used and their data sources.

| Variables | | Description | Unit | Source reference |
| --- | --- | --- | --- | --- |
| Health | Malaria prevalence | Presence of parasitemia using blood smear test under microscopy | 1/0 | (ICF, 2017) |
| Environmental /Ecological | Deforestation | Change in vegetation if greater than 5% between the years 1992/93 and 2010/11. Forest cover was measured as proportion of area within 5 km buffer around each cluster in each year. | % | (DeFries et al., 2000; DiMiceli et al., 2011) |
|  | *Anopheles gambiae* | The predicted probability of occurrence *An. gambiae* in a sampling cluster sites (binary variable, if the probability is > 50%, present =1 otherwise 0) | 1/0 | (Wiebe et al., 2017) |
|  | *Anopheles arabiensis* | The predicted probability of occurrence *An. arabiensis* in a sampling cluster sites (binary variable, if the probability is > 50%, present =1 otherwise 0) | 1/0 | (Wiebe et al., 2017) |
|  | *Anopheles funestus* | The predicted probability of occurrence *An. funestus* in a sampling cluster sites (binary variable, if the probability is > 50%, present =1 otherwise 0) | 1/0 | (Wiebe et al., 2017) |
| Demographic | Population density | Average population density within 5 km buffer around cluster for the survey years | Individuals per km^2^ | (CIESIN, 2016) |
|  | Livestock density | Ruminant livestock density in the 10 km cluster buffer in Tropical Livestock Unit (TLU) per km^2^ categorized: (1= less than 1; 2=1-5; 3= 5-10; 4=: 10-20; 5: 20-50; 6=50-100; 7=100-200; 8: >= 200) | TLU, Categorical | (FAO/IIASA, 2010) |
|  | Age of a child | Age of a child in months at the time of survey | Months | (ICF, 2017) |
|  | Rural residence | Whether the household residence is rural or urban areas (rural =1, urban=0) | 1/0 | (ICF, 2017) |
| Socioeconomic | Poorest | The first quintile of the wealth index | 1/0 | (ICF, 2017) |
|  | Poorer | The second quintile of the wealth index | 1/0 | (ICF, 2017) |
|  | Middle  Richer | The Middle quintile of the wealth index  The fourth quintile of the wealth index | 1/0  1/0 | (ICF, 2017)  (ICF, 2017) |
|  | Richest | The fifth quintile of the wealth index | 1/0 | (ICF, 2017) |
|  | Bed net use | Whether all or some of the children within a household slept in an insecticide treated bed net the night before the survey (1= yes, 0 for ‘none’) | 1/0 | (ICF, 2017) |
| Climate | Average annual temperature | Long-term (30 year) average monthly mean temperature at the cluster site during the survey month | ^o^C | (Hijmans et al., 2005) |
|  | Wettest quarter precipitation | Total monthly rainfall in the cluster for the wettest quarter | mm | (Hijmans et al., 2005) |

Table S2. Summary of country level DHS sample population by survey year

| Country | Survey year | No. of clusters | No of Children |
| --- | --- | --- | --- |
| Democratic Republic of the Congo | 2013/14 | 233 | 2982 |
| Cote d’Ivore | 2011/12 | 108 | 1036 |
| Guinea | 2012 | 99 | 1104 |
| Mozambique | 2011 | 281 | 2384 |
| Rwanda | 2010/11 | 255 | 1890 |
| Togo | 2013/14 | 257 | 2350 |
| Total |  | **1233** | **11746** |

Table S3. Descriptive summary of variables by study countries and the overall average

| Variables | | Unit | Democratic Republic of the Congo | | Rwanda | | Mozambique | | Cote d’Ivore | | Guinea | | Togo | | Overall average | |
| --- | --- | --- | --- | --- | --- | --- | --- | --- | --- | --- | --- | --- | --- | --- | --- | --- |
|  |  |  | Mean | SD | Mean | SD | Mean | SD | Mean | SD | Mean | SD | Mean | SD | **Mean** | **SD** |
| Health | Malaria prevalence | % | 27.06 | 44.44 | 1.8 | 13.29 | 38.1 | 48.6 | 14.86 | 35.56 | 54.98 | 49.77 | 37.6 | 48.4 | **28.9** | **45.3** |
| Environmental /Ecological | Deforestation | % | 22.68 | 24.99 | 8.26 | 9.8 | 49.94 | 32.6 | 8.09 | 15.35 | 13.55 | 20.76 | 2.31 | 9.16 | **19.69** | **27.46** |
|  | *Anopheles gambiae* occurrence | % | 68.15 | 46.6 | 99.1 | 9.44 | 10.71 | 30.9 | 94.01 | 23.73 | 76.08 | 42.67 | 87.73 | 32.82 | **68.48** | **46.46** |
|  | *Anopheles arabiensis* occurrence | % | 75.02 | 43.29 | 60.68 | 48.86 | 15.52 | 36.2 | 5.50 | 22.81 | 10.23 | 30.32 | 11.97 | 32.47 | **35.71** | **47.91** |
|  | *Anopheles funestus* occurrence | % | 21.47 | 41.07 | 13.95 | 34.66 | 95.02 | 21.7 | 34.75 | 47.64 | 87.13 | 33.49 | 23.95 | 42.69 | **43.01** | **49.51** |
| Demographic | Population density | Per km^2^ | 1972 | 5652 | 999.2 | 1331 | 149.7 | 349 | 56.02 | 49.88 | 74.74 | 132.1 | 896.3 | 1847 | **885.4** | **3113** |
|  | Livestock density | TLU | 1.76 | 0.94 | 5.31 | 0.86 | 1.84 | 1.27 | 2.78 | 1.43 | 3.37 | 0.99 | 4.35 | 1.72 | **3.10** | **1.85** |
|  | Age of a child | months | 31.4 | 15.67 | 32.6 | 15.4 | 30.74 | 15.5 | 30.76 | 15.29 | 31.36 | 15.69 | 31.3 | 15.5 | **31.38** | **15.55** |
|  | Rural residence | % | 64.35 | 47.90 | 78.01 | 41.43 | 81.01 | 39.23 | 66.85 | 47.32 | 86.05 | 34.66 | 74.61 | 43.53 | **74.20** | **43.75** |
| Socioeconomic | Poorest | % | 20.12 | 40.14 | 18.71 | 39.01 | 23.88 | 42.63 | 23.07 | 42.14 | 40.39 | 49.27 | 36.94 | 48.28 | **26.31** | **44.03** |
|  | Poorer | % | 22.63 | 41.85 | 17.55 | 38.05 | 25.97 | 43.87 | 16.41 | 37.05 | 23.28 | 42.28 | 20.42 | 40.32 | **21.55** | **41.11** |
|  | Middle  Richer | %  % | 20.86  19.85 | 40.64  39.82 | 17.78  20.08 | 38.25  40.07 | 24.11  17.94 | 42.78  38.35 | 29.63  15.54 | 45.68  36.25 | 16.75  16.03 | 37.36  36.70 | 15.83  13.8 | 36.50  34.5 | **20.40**  **17.53** | **40.30**  **38.02** |
|  | Richest | % | 16.72 | 37.32 | 25.85 | 43.79 | 8.11 | 27.28 | 15.34 | 36.06 | 2.54 | 15.73 | 12.9 | 33.58 | **14.21** | **34.91** |
|  | Bed net use | % | 56.59 | 49.57 | 71.99 | 44.92 | 33.75 | 47.28 | 39.48 | 48.90 | 37.41 | 48.41 | 45.9 | 49.85 | **49.08** | **49.99** |
| Climate | Average annual temperature | ^o^C | 30.6 | 1.08 | 27.02 | 0.69 | 30.31 | 1.95 | 32.56 | 0.49 | 33.45 | 1.33 | 33.15 | 0.97 | **30.91** | **2.43** |
|  | Wettest quarter precipitation | mm | 593.9 | 71.1 | 395.1 | 50.9 | 649.3 | 143.4 | 581.9 | 137.1 | 949.4 | 211.5 | 587.5 | 100.4 | **604.1** | **180.3** |

Table S4. Summary of the variables by different wealth quintiles for the study countries

| **Variables** | **Poorest (n=3090)** | | **Poorer (n=2531)** | | **Middle**  **(n=2397)** | | **Richer**  **(n=2059)** | | **Richest**  **(n=1669)** | |
| --- | --- | --- | --- | --- | --- | --- | --- | --- | --- | --- |
|  | *Mean* | *SD* | *Mean* | *SD* | *Mean* | *SD* | *Mean* | *SD* | *Mean* | *SD* |
| Malaria prevalence (%) | 40.42 | 49.08 | 36.66 | 48.19 | 28.78 | 45.29 | 20.45 | 40.34 | 6.23 | 24.18 |
| Deforestation (%) | 20.47 | 28.05 | 24.91 | 29.73 | 24.97 | 30.39 | 16.33 | 24.01 | 6.87 | 14.66 |
| Population density (per km2) | 115.6 | 176.8 | 116.6 | 184.9 | 189.32 | 473.47 | 950.2 | 2512.2 | 4396.4 | 6706.3 |
| Livestock density (TLU) | 3.067 | 1.77 | 2.73 | 1.68 | 2.91 | 1.75 | 3.34 | 1.9 | 3.76 | 2.08 |
| Age of a child (months) | 31.48 | 15.61 | 31.28 | 15.47 | 31.2 | 15.57 | 31.58 | 15.53 | 31.36 | 15.55 |
| Rural residence (%) | 94.24 | 23.3 | 96.13 | 19.29 | 86.06 | 34.64 | 51.91 | 49.97 | 14.32 | 35.04 |
| Bed net use (%) | 44.76 | 49.73 | 48.04 | 49.97 | 49.10 | 50.00 | 51.09 | 50 | 56.2 | 49.63 |
| Average annual temperature (oC) | 31.76 | 2.47 | 30.98 | 2.29 | 30.79 | 2.27 | 30.56 | 2.37 | 29.86 | 2.3 |
| Wettest quarter precipitation (mm) | 642.5 | 163.1 | 618.3 | 168.8 | 606.29 | 179.09 | 584.3 | 186.31 | 532.95 | 197.4 |

Table S5. Summary of multilevel mixed effects model results (coefficients) by SSA regions

| Variables | Central & Eastern Africa | South Africa | West Africa |
| --- | --- | --- | --- |
|  | ***Coef. (Std.err)*.** | ***Coef. (Std.err)*** | ***Coef. (Std.err)*.** |
| Deforestation | 0.252* | 0.191* | 0.221 |
|  | (0.104) | (0.0892) | (0.13) |
| Population density | 0.000 | 0.000 | 0.000 |
|  | (0.000) | (0.000) | (0.000) |
| Livestock density (TLU) | -0.306** | -0.015 | -0.174** |
|  | (0.106) | (0.101) | 0.064 |
| Age of a child (months) | 0.391*** | 0.392*** | 0.351*** |
|  | (0.054) | (0.067) | (0.043) |
| Rural | -0.162 | 0.319 | 1.257*** |
|  | (0.299) | (0.365) | (0.266) |
| Poorest | 0.327 | 0.435* | 0.451** |
|  | (0.18) | (0.22) | (0.148) |
| Poorer | 0.293 | 0.102 | 0.25 |
|  | (0.17) | (0.206) | (0.151) |
| Richer | -0.0811 | -0.714** | -0.175 |
|  | (0.196) | (0.258) | (0.192) |
| Richest | -0.913** | -1.497** | -1.040** |
|  | (0.317) | (0.457) | (0.318) |
| Bed net use | -0.460*** | 0.0406 | -0.337*** |
|  | (0.119) | (0.159) | (0.096) |
| Average annual temperature (ºC) | 1.153*** | 0.919*** | 0.087 |
|  | (0.256) | (0.198) | (0.254) |
| Wettest quarter precipitation (mm) | 0.411 | 0.879*** | 0.181** |
|  | (0.22) | (0.17) | (0.062) |
| Constant | 0.174 | -0.896 | -1.275*** |
|  | (0.389) | (0.483) | (0.326) |
| *Random intercept variances* |  |  |  |
| Cluster | 2.442*** | 1.888*** | 1.872*** |
|  | (0.381) | (0.375) | (0.255) |
| Households | 0.574* | 1.914*** | 0.715** |
|  | (0.27) | (0.544) | (0.224) |
| No. of observations | 4872 | 2384 | 4490 |
| Log-likelihood | -1370.678 | -1294.692 | -2402.705 |
| Prob > chi2 | 0.0000 | 0.0000 | 0.0000 |

Significance levels: *** p<0.001, ** p<0.01, * <0.05. Standard errors in parentheses

Table S6. Summary of multilevel mixed effects model results (coefficients) by wealth levels

| Variables | Poorest | Poorer | Middle | Richer | Richest |
| --- | --- | --- | --- | --- | --- |
|  | ***Coef. (Std.err)*** | ***Coef. (Std.err)*** | ***Coef. (Std.err)*** | ***Coef. (Std.err)*** | ***Coef. (Std.err)*** |
| Deforestation | 0.259*** | 0.284*** | 0.273*** | 0.240* | 0.181 |
|  | (0.078) | (0.079) | (0.078) | (0.094) | (0.264) |
| Population density | -0.001 | 0.0006 | 0.0002 | -0.000 | 0.000 |
|  | (0.0008) | (0.0006) | (0.0002) | (0.000) | (0.000) |
| Livestock density (TLU) | -0.218** | -0.255*** | -0.288*** | -0.271*** | -0.217* |
|  | (0.069) | (0.073) | (0.069) | (0.064) | (0.101) |
| Age of a child (months) | 0.355*** | 0.449*** | 0.357*** | 0.315*** | 0.281 |
|  | (0.052) | (0.065) | (0.065) | (0.077) | (0.143) |
| Rural | -0.53 | 0.372 | 0.432 | 0.358 | 0.267 |
|  | (0.355) | (0.427) | (0.276) | (0.222) | (0.63) |
| Bed net use | -0.303* | -0.206 | -0.526*** | -0.408* | -0.707* |
|  | (0.12) | (0.142) | (0.146) | (0.171) | (0.352) |
| Average annual temperature (^o^C) | 0.816*** | 0.988*** | 0.959*** | 0.780*** | 0.961** |
|  | (0.147) | (0.148) | (0.151) | (0.152) | (0.294) |
| Wettest quarter precipitation (mm) | 0.435*** | 0.567*** | 0.382*** | 0.316*** | 0.0818 |
|  | (0.094) | (0.101) | (0.087) | (0.084) | (0.144) |
| Constant | 0.786* | -0.288 | -0.348 | -0.735** | -2.723*** |
|  | (0.387) | (0.455) | (0.322) | (0.277) | (0.624) |
| *Random intercept Variances* |  |  |  |  |  |
| Cluster | 2.290*** | 2.245*** | 2.219*** | 1.297*** | 1.292 |
|  | (0.352) | (0.401) | (0.405) | (0.366) | (0.861) |
| Households | 0.613* | 1.156** | 0.709 | 1.448* | 4.554* |
|  | (0.261) | (0.432) | (0.378) | (0.579) | (2.203) |
| No. of observations | 3090 | 2531 | 2397 | 2059 | 1669 |
| Log-likelihood | -1722.17 | -1370.68 | -1214.63 | -904.64 | -350.88 |
| Prob > chi2 | 0.0000 | 0.0000 | 0.0000 | 0.0000 | 0.0000 |

Significance levels: *** p<0.001, ** p<0.01, * <0.05. Standard errors in parentheses

Table S7. Summary of multilevel mixed effects model results (coefficients) by dominant vector species

| Variables | *Anopheles gambiae* | *An. arabiensis* | *An. funestus* |
| --- | --- | --- | --- |
|  | ***Coef. (Std.err)*** | ***Coef. (Std.err)*** | ***Coef. (Std.err)*** |
| Deforestation | 0.286*** | 0.228 | 0.228*** |
|  | (0.080) | (0.128) | (0.067) |
| Population density | 0.0000 | -0.0000 | -0.0002 |
|  | (0.000) | (0.000) | (0.0003) |
| Livestock density (TLU) | -0.341*** | -0.284*** | -0.201** |
|  | (0.049) | (0.068) | (0.063) |
| Age of a child (months) | 0.364*** | 0.344*** | 0.381*** |
|  | (0.037) | (0.053) | (0.043) |
| Rural | 0.579** | -0.283 | 0.446 |
|  | (0.217) | (0.279) | (0.261) |
| Poorest | 0.359** | 0.420* | 0.425** |
|  | (0.123) | (0.184) | (0.136) |
| Poorer | 0.175 | 0.176 | 0.109 |
|  | (0.123) | (0.182) | (0.134) |
| Richer | -0.151 | -0.0375 | -0.403* |
|  | (0.149) | (0.204) | (0.167) |
| Richest | -0.878*** | -0.916** | -1.461*** |
|  | (0.241) | (0.321) | (0.323) |
| Bed net use | -0.383*** | -0.373** | -0.174 |
|  | (0.081) | (0.12) | (0.096) |
| Average annual temperature (^o^C) | 1.132*** | 0.969*** | 0.649*** |
|  | (0.113) | (0.16) | (0.122) |
| Wettest quarter precipitation (mm) | 0.294*** | 0.419* | 0.449*** |
|  | (0.066) | (0.163) | (0.069) |
| Constant | -0.265 | 0.0174 | -0.794* |
|  | (0.255) | (0.367) | (0.32) |
| *Random intercept Variances* |  |  |  |
| Cluster | 2.350*** | 2.168*** | 2.163*** |
|  | (0.255) | (0.351) | (0.274) |
| Households | 0.710*** | 0.673* | 0.878*** |
|  | (0.19) | (0.284) | (0.237) |
| No. of observations | 8044 | 4195 | 5052 |
| Log-likelihood | -3475.23 | -1607.82 | -2676.77 |
| Prob > chi2 | 0.0000 | 0.0000 | 0.0000 |

Significance levels: *** p<0.001, ** p<0.01, * <0.05. Standard errors in parentheses

Data Set S1. Dataset used for analysis is available and archived in Figshare open access repository (Estifanos *et al*., 2024).

Data Set S2. Stata coded used for modeling is available and archived in Figshare open access repository (Estifanos *et al*., 2024).

Akpan, G. E., Adepoju, K. A., Oladosu, O. R., & Adelabu, S. A. (2018). Dominant malaria vector species in Nigeria: Modelling potential distribution of Anopheles gambiae sensu lato and its siblings with MaxEnt. *PLOS ONE*, *13*(10), e0204233. <https://doi.org/10.1371/journal.pone.0204233>

CIESIN. (2016). Center for International Earth Science Information Network - CIESIN - Columbia University. 2016. Gridded Population of the World, Version 4 (GPWv4): Population Count Adjusted to Match 2015 Revision of UN WPP Country Totals. Palisades, NY: NASA Socioeconomic Data and Applications Center (SEDAC) [Dataset] <http://dx.doi.org/10.7927/H4HX19NJ>.

DeFries, R. S., Hansen, M. C., Townshend, J. R. G., Janetos, A. C., & Loveland, T. R. (2000). 1 kilometer tree cover continuous fields, 1.0, Department of Geography, University of Maryland, College Park, Maryland, 1992–1993. [Dataset] <http://www.landcover.org/data/treecover/>.

DiMiceli, C. M., Carroll, M. L., Sohlberg, R., Huang, C., Hansen, M. C., & Townshend, J. R. (2011). *Annual Global Automated MODIS Vegetation Continuous Fields (MOD44B) at 250 m Spatial Resolution for Data Years Beginning Day 65, 2000 - 2010* Collection 5 Percent Tree Cover, University of Maryland, College Park, USA. [Dataset] http://www.landcover.org/data/vcf/

Estifanos, T.K., Fisher, B., Gillian L. Galford, &amp; Ricketts, T., (2024). Deforestation effect on childhood malaria depends on vectors biology and wealth. [Dataset] DOI: 10.6084/m9.figshare.25067678

FAO/IIASA. (2010). Global Agro-ecological Zones (GAEZ v3.0). FAO, Rome, Italy and IIASA, Laxenburg, Austria. [Dataset] [http://gaez.fao.org/Main.html#](http://gaez.fao.org/Main.html). Accessed 02/01/2017.

Galford, G. L., Soares-Filho, B. S., Sonter, L. J., & Laporte, N. (2015). Will Passive Protection Save Congo Forests? *PLOS ONE*, *10*(6), e0128473. <https://doi.org/10.1371/journal.pone.0128473>

Hansen, M. C., DeFries, R. S., Townshend, J. R. G., Carroll, M., Dimiceli, C., & Sohlberg, R. A. (2003). Global Percent Tree Cover at a Spatial Resolution of 500 Meters: First Results of the MODIS Vegetation Continuous Fields Algorithm. *Earth Interactions*, *7*(10), 1-15. <https://doi.org/10.1175/1087-3562(2003)007><0001:GPTCAA>2.0.CO;2

Hijmans, R. J., Cameron, S. E., Parra, J. L., Jones, P. G., & Jarvis, A. (2005). Very high resolution interpolated climate surfaces for global land areas [10.1002/joc.1276]. *International Journal of Climatology*, *25*(15), 1965-1978. <https://doi.org/https://doi.org/10.1002/joc.1276>

ICF. (2012). *Demographic and Health Survey Sampling and Household Listing Manual. MEASURE DHS, Calverton, Maryland, U.S.A.: ICF International*. [Dataset] <https://dhsprogram.com/publications/publication-dhsm4-dhs-questionnaires-and-manuals.cfm>

ICF. (2017). 2004-2017. Demographic and Health Surveys (DHS). [Various Datasets] Funded by USAID. Rockville, Maryland: ICF International. Available at <www.dhsprogram.com>.

Leyland, A. H., & Groenewegen, P. P. (2020). *Multilevel modelling and public health and health services research*. Springer, Switzerland.

Mitchell, C. L., Janko, M. M., Mwandagalirwa, M. K., Tshefu, A. K., Edwards, J. K., Pence, B. W., Juliano, J. J., & Emch, M. (2022). Impact of extractive industries on malaria prevalence in the Democratic Republic of the Congo: a population-based cross-sectional study. *Scientific Reports*, *12*(1), 1737. <https://doi.org/10.1038/s41598-022-05777-9>

Potapov, P. V., Turubanova, S. A., Hansen, M. C., Adusei, B., Broich, M., Altstatt, A., Mane, L., & Justice, C. O. (2012). Quantifying forest cover loss in Democratic Republic of the Congo, 2000–2010, with Landsat ETM+ data. *Remote Sensing of Environment*, *122*, 106-116. [Dataset] <https://doi.org/10.1016/j.rse.2011.08.027>

Shah, H. A., Carrasco, L. R., Hamlet, A., & Murray, K. A. (2022). Exploring agricultural land-use and childhood malaria associations in sub-Saharan Africa. *Scientific Reports*, *12*(1), 4124. <https://doi.org/10.1038/s41598-022-07837-6>

Sinka, M. E., Bangs, M. J., Manguin, S., Coetzee, M., Mbogo, C. M., Hemingway, J., Patil, A. P., Temperley, W. H., Gething, P. W., Kabaria, C. W., Okara, R. M., Van Boeckel, T., Godfray, H. C. J., Harbach, R. E., & Hay, S. I. (2010). The dominant Anopheles vectors of human malaria in Africa, Europe and the Middle East: occurrence data, distribution maps and bionomic précis. *Parasites & Vectors*, *3*(1), 117. <https://doi.org/10.1186/1756-3305-3-117>

Wiebe, A., Longbottom, J., Gleave, K., Shearer, F. M., Sinka, M. E., Massey, N. C., Cameron, E., Bhatt, S., Gething, P. W., Hemingway, J., Smith, D. L., Coleman, M., & Moyes, C. L. (2017). Geographical distributions of African malaria vector sibling species and evidence for insecticide resistance. *Malaria Journal*, *16*(1), 85. [Dataset] <https://doi.org/10.1186/s12936-017-1734-y>
